# Clinical and demographic associations of recorded feigning in functional neurological disorder

**DOI:** 10.1093/braincomms/fcaf490

**Published:** 2025-12-12

**Authors:** Rok Berlot, Thomas A Pollak, Livia Asan, Biba Stanton, Timothy R Nicholson, Mark J Edwards, Richard A Kanaan

**Affiliations:** Department of Basic & Clinical Neuroscience, Institute of Psychiatry, Psychology & Neuroscience, King’s College London, London SE5 8AB, UK; Department of Neurology, University Medical Centre Ljubljana, Ljubljana 1000, Slovenia; Faculty of Medicine, University of Ljubljana, Ljubljana 1000, Slovenia; Neuropsychiatry Research & Education Group, Institute of Psychiatry, Psychology & Neuroscience, King’s College London, London SE5 8AB, UK; Department of Psychosis Studies, Institute of Psychiatry, Psychology & Neuroscience, King’s College London, London SE5 8AB, UK; Department of Basic & Clinical Neuroscience, Institute of Psychiatry, Psychology & Neuroscience, King’s College London, London SE5 8AB, UK; Department of Neurology, Center for Translational and Behavioral Neurosciences, University Hospital Essen, Essen 45147, Germany; Department of Neurology, King’s College Hospital NHS Foundation Trust, London SE5 9RS, UK; Neuropsychiatry Research & Education Group, Institute of Psychiatry, Psychology & Neuroscience, King’s College London, London SE5 8AB, UK; Department of Basic & Clinical Neuroscience, Institute of Psychiatry, Psychology & Neuroscience, King’s College London, London SE5 8AB, UK; Department of Neuropsychiatry, South London and Maudsley NHS Foundation Trust, London SE5 8AZ, UK; Department of Psychiatry, University of Melbourne, Austin Health, Heidelberg 3084, Australia; The Florey Institute of Neuroscience and Mental Health, Heidelberg 3084, Australia

**Keywords:** functional neurological disorder, conversion disorder, malingering, factitious disorder, clinician bias

## Abstract

Although functional neurological disorder (FND) is common, increasingly recognized, potentially disabling, and treatable, it remains stigmatized, and concerns about feigning persist among clinicians. We examined the prevalence of malingering and factitious disorder diagnoses in individuals with FND, their associated demographic and clinical characteristics, and evidence of clinician bias in the diagnosis of feigning. In this retrospective cohort and case-control study using the international TriNetX electronic health record network, we analysed diagnostic codes (International Classification of Diseases, Tenth Revision) for FND, malingering and factitious disorder to assess their prevalence and overlap. We then compared rates of malingering and factitious disorder following a diagnosis of FND with those in cohorts of patients with multiple sclerosis and with depression, used as comparison conditions. We also examined demographic characteristics and comorbidities of FND cases with and without records of feigning, as well as temporal trends in the proportion diagnosed with malingering. Between 2015 and 2024, 143 471 individuals were diagnosed with FND, 54 685 with malingering and 5215 with factitious disorder. 2.2% of individuals with FND also received a record of malingering or, less commonly, factitious disorder, or both. Following diagnosis, FND was associated with higher rates of malingering (1.36%) and factitious disorder (0.62%) compared to multiple sclerosis (0.17%, odds ratio 7.97, 95% confidence interval 7.17–8.87; and 0.03%, odds ratio 20.47, 95% confidence interval 16.15–25.94, respectively) and depression (0.42%, odds ratio 3.23, 95% confidence interval 3.08–3.39; and 0.05%, odds ratio 12.17, 95% confidence interval 11.18–13.31, respectively). Among FND cases, factitious disorder was more prevalent in White individuals, whereas malingering was more frequent in males, Black individuals and seizure presentations. Compared to other FND cases, those with diagnoses of malingering or factitious disorder had more psychiatric, neurological, and medical comorbidities, greater socio-economic adversity and increased mortality. Records of malingering were more likely in FND cases with histories of other stigmatized disorders, such as sexually transmitted diseases, viral hepatitis and HIV. Their proportion declined from 2018 to 2023. Malingering and factitious disorder are more frequently diagnosed in FND than in comparable disorders, although both remain uncommon. Their presence is associated with greater clinical complexity and poorer outcomes. Associations with ethnicity, socio-economic adversity and certain comorbidities suggest possible clinician bias, while declining malingering diagnoses in FND may reflect growing awareness among clinicians.

## Teaser

Analysing electronic health records, Berlot et al. found 2.2% of functional neurological disorder cases also had recorded malingering or factitious disorder. These individuals had more comorbidities, with higher malingering rates among Black patients and those with stigmatised conditions, suggesting clinician bias and underscoring the need for more informed, equitable care.

## Graphical Abstract

**Graphical Abstract:**
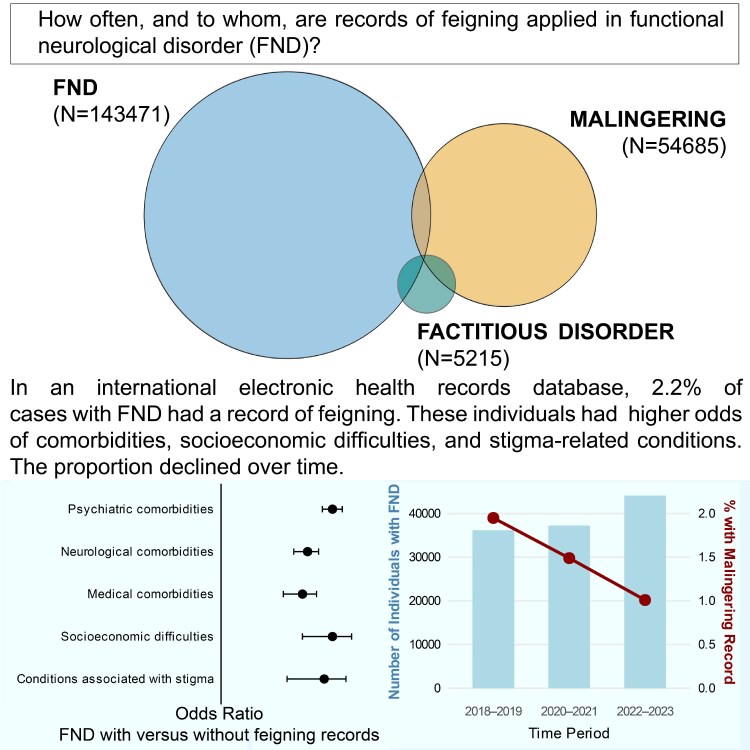

## Introduction

Functional neurological disorder (FND) is a common condition with diverse presentations, including seizures, motor, sensory and cognitive symptoms.^1^ It often affects younger individuals, may lead to impaired quality of life and lifelong disability, and incurs substantial health and social care costs.^2^ It is also increasingly recognized as treatable. However, it remains, to a large extent, a neglected condition, and individuals with FND often face outdated misconceptions and attitudes in clinical settings.^3^ Questions regarding the voluntariness of symptoms often lie at the core of these biases.^4,5^

The diagnostic approach to FND, based on positive features rather than exclusion, has become increasingly familiar to clinicians. To a large extent, this relies on demonstrating preserved capacity for normal function, indicating that symptoms arise from impaired access to, or control over, normal bodily function.^6^ However, they often remain concerned about the possibility of deliberate symptom fabrication, whether for external gain, as seen in malingering, or care-seeking, as in factitious disorder.^7,8^ This may, in part, stem from the involvement of the voluntary motor or sensory system in symptom generation. Although experienced as involuntary, symptoms of FND often phenomenologically resemble voluntary actions.^9^

A considerable proportion of neurologists and psychiatrists consider feigning to be relatively common in FND, perhaps as part of an overlapping continuum with FND or as a subset of the same phenomenon.^10-13^ An alternative perspective, embraced by many clinicians treating FND, is that factitious disorder and malingering are clearly distinct from FND. This view is supported by, among others, the consistency of FND presentations across time and cultures, consistency in individuals’ subjective illness experiences and responses to therapeutic interventions, as well as behavioural and neuroimaging studies.^4^ This does not exclude the possibility that feigning or symptom exaggeration may appear as an additional phenomenon in people with FND, and their feeling of not being taken seriously may contribute to such behaviour.^4^ However, deception is not unique to FND, as it also occurs among the general population for various gains.^14,15^ In addition, aggravation of symptoms, as well as increased self-reporting of symptoms and disability driven by health-related anxiety, are also observed in other medical conditions, such as multiple sclerosis (MS).^16^

A longstanding challenge in studying feigning has been the limited access to confirmed clinical cases, resulting in much of the literature being largely speculative. Most physicians encounter very few individuals they can confidently identify as feigning illness, and even fewer who are willing to participate in research. More recently, the advent of electronic health records has enabled reporting of larger samples.^17-20^ However, the relationship between malingering, factitious disorder and FND has not been explored at scale.

Utilizing TriNetX, a large international electronic health records network, we investigated the frequency of malingering and factitious disorder diagnoses in individuals with FND and explored potential risk factors for receiving this diagnosis, including demographic variables and common neurological, psychiatric and medical comorbidities. Specifically, we: (i) assessed the base rates and overlap of FND, malingering and factitious disorder; (ii) compared rates of feigning records in FND versus MS and depression to determine if FND increases the likelihood of these diagnoses; (iii) compared demographic and clinical characteristics of individuals with FND who had received a diagnosis of malingering or factitious disorder to those who had not; (iv) investigated variables potentially linked to clinician bias, given the stigma surrounding FND^21^ and prior concerns about biases in malingering diagnoses;^17^ and (v) analysed trends over time in proportion of FND cases with records of malingering to assess potential shifts in clinician attitudes, perhaps influenced by increased education.^22^

## Materials and methods

### Data and study design

The study was conducted using TriNetX, a global federated health research network that compiles de-identified electronic health record data from over 164 million patients. To comply with legal and ethical standards, the identities of individual contributing healthcare organizations (which include hospitals, specialist providers and primary care facilities) and their specific data contributions are not disclosed. Cohorts are generated via the TriNetX user interface by applying inclusion and exclusion criteria. In response to centralized queries, results are collected and aggregated. Data are standardized using recognized clinical terminologies, which include demographics (coded according to HL7 standards) and diagnoses (represented by ICD-10-CM, the International Classification of Diseases, Tenth Revision, Clinical Modification), procedures and measurements (additional information about the TriNetX network is provided within Supplementary Methods).

This retrospective study is exempt from obtaining informed consent, and institutional ethical approval was not required. The data analysed are secondary data, do not involve intervention or interaction with human subjects, and have been de-identified in line with the de-identification standard outlined in Section §164.514(b)(1) of the HIPAA Privacy Rule (http://trinetx.com). The RECORD reporting guidelines were followed. The analyses were performed in May and June 2025.

### Prevalence and overlap of FND, malingering and factitious disorder

Individuals aged 13 to 80 who received a new diagnosis of FND between 1 January 2015 and 31 December 2024 were included. The diagnosis of FND was defined using ICD-10 codes commonly used for typical phenotypes: conversion disorder with motor symptom or deficit (F44.4), conversion disorder with seizures or convulsions (F44.5), conversion disorder with sensory symptom or deficit (F44.6), conversion disorder with mixed symptom presentation (F44.7), other dissociative and conversion disorders (F44.89), dissociative and conversion disorder, unspecified (F44.9). These are most commonly used to code for the typical phenotypes of FND: functional motor disorder, functional/dissociative seizures, functional sensory disorder, FND with mixed symptoms and other or unspecified FND. We excluded other F44 diagnostic category codes, such as dissociative fugue, amnesia, stupor and identity disorder, as these are not typically used for the most common FND phenotypes. In addition, cohorts with new records of ‘Malingerer (conscious simulation)’ (Z76.5) or ‘Factitious disorder imposed on self’ (F68.1) during the same period were constructed. Cases with records of conversion and dissociative disorders (F44), other disorders of adult personality and behaviour (F68) and malingering (Z76.5) before the observation period were excluded. We assessed the prevalence and overlap of FND, malingering and factitious disorder records during the observation period.

### Rates of malingering and factitious disorder in FND, MS and depression

To determine if the FND diagnosis increases the likelihood of a subsequent record of feigning, the FND cohort, described above, was compared to a contemporaneous cohort of individuals with newly diagnosed MS (G35), a condition with similar prevalence, female predominance, age distribution and comparable disability.^23,24^ Two outcomes were assessed after the diagnosis of each condition: a new diagnosis of malingering and that of factitious disorder. We compared these outcomes between cohorts with FND and MS. An analogous analysis was performed comparing individuals with FND to a cohort with a diagnosis of ‘Major depressive disorder, recurrent’ (F33).

As sensitivity analyses, we compared cohorts matched for demographic variables and records of socio-economic difficulties, as well as cohorts excluding individuals diagnosed with both MS and FND, or with diagnoses of both FND and depression (Supplementary Methods).

### Demographics, clinical features and comorbidities in FND with and without a record of malingering or factitious disorder

Two comparisons were made among individuals with FND: those without a record of factitious disorder or malingering were compared to (i) those with malingering and (ii) those with factitious disorder, recorded at any point during the observation period. We compared demographic variables, rates of distinct FND subtypes, psychiatric, neurological and common medical comorbidities, socio-economic adversity using codes for obesity, malnutrition, problems related to education and literacy, employment and unemployment and housing and economic circumstances. We also compared the rates of wheelchair use and mortality. The binary variables were considered present if they appeared at any time in the electronic health records.

### Comorbidities associated with stigma in malingering

Previous research suggests that racial biases may influence the application of the malingering label,^17,25^ and malingering has been associated with more negative clinician attitudes than FND.^26^ We therefore aimed to explore additional sources of stigma that may be at play, particularly those arising from comorbid diagnoses. These could lead to overdiagnosis of malingering. We identified variables that may contribute additional layers of stigma by biasing clinicians’ judgements based on (i) a person’s visible traits, (ii) perceived personal responsibility and moral judgement or (iii) an association with socially sensitive topics. We assessed whether they are related to an increased relative risk (RR) of a recorded code of malingering in individuals with FND. However, one hypothesis is that medical comorbidities lead to more frequent medical encounters, increasing the likelihood of FND being labelled as malingering. To distinguish this potential effect from that of clinician bias, we included comparison conditions with overlapping clinical features and similar follow-up needs, but which are less commonly associated with stigma.

We assessed three categories of conditions: (i) the visible skin conditions psoriasis and atopic dermatitis;^27^ (ii) infections associated with stigma including sexually transmitted diseases (STD), compared to urinary tract infections (UTI); viral hepatitis, compared to non-alcoholic fatty liver; and HIV, hepatitis B and hepatitis C;^28^ and (iii) among individuals with a diagnosis of post-traumatic stress disorder (PTSD), we compared those with and without a recorded history of adult or child abuse, neglect or maltreatment, a risk factor for the development of FND in some patients.^29,30^

### Changing attitudes toward malingering and FND

As the ascription of malingering labels to FND is associated with the level of education of the ascriber,^22^ changes in their use may reflect shifting attitudes toward FND. To test this, we examined the prevalence of malingering records within a year before or after any recorded FND diagnosis across 3 two-year periods (2018–2019, 2020–2021 and 2022–2023). For each cohort, we calculated the proportion of individuals with a record of malingering within one year before or after any recorded diagnosis of FND.

### Statistical analysis

The demographic features of individuals with records of FND, malingering and factitious disorder were analysed using the statistical tools available within the TriNetX platform. Age (two-tailed *t*-test), gender and race (two-tailed z-tests) were compared, with significance set at *P* < 0.005 (applying Bonferroni correction for 10 variables per comparison). Similarly, demographic variables, clinical features and comorbidities of individuals with FND without records of feigning were compared to those with a record of malingering or factitious disorder. Statistical significance was set at *P* < 0.0011 (applying Bonferroni correction for 45 variables per comparison). Records of malingering and factitious disorder were compared as outcomes following a diagnosis of FND and MS or depression using the measure of association analysis within the TriNetX platform. Odds ratios (OR) were calculated with significance set at *P* < 0.025 (applying Bonferroni correction for two outcomes).

In the analysis investigating the association between comorbidities with stigma and records of malingering in FND, the RR for a record of malingering stratified by each condition was calculated using *R* (version 4.5.0) and represented using forest plots. Statistical significance was set at *P* < 0.0045 (applying Bonferroni correction for 11 conditions assessed). Using *R*, a Chi-squared test of independence was performed to explore differences in malingering rates across cohorts corresponding to different time periods.

## Results

### Prevalence of FND, malingering and factitious disorder

**Figure 1:**
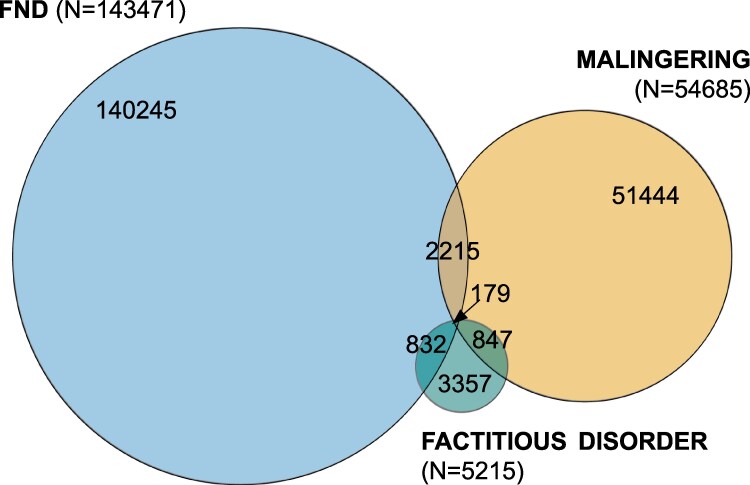
**Prevalence and overlap of FND, malingering and factitious disorder.** The counts in the Venn diagram correspond to single patients with recorded FND, malingering and/or factitious disorder.

Between 1 January 2015 and 31 December 2024, 143 471 individuals received a new diagnosis of FND, 54 685 of malingering and 5215 of factitious disorder. Overlap between diagnoses was observed: 1.5% of FND cases had an additional diagnosis of malingering only, 0.6% had an additional diagnosis of factitious disorder only and 0.1% had both of these additional diagnoses (Fig. 1).

Compared to people with FND, individuals with malingering or factitious disorder were, on average, older (Table 1). The proportion of males was higher in malingering (58%) and factitious disorder (34%) compared to FND (29%). Racial differences were observed, with the Black or African American race being more common in malingering and factitious disorder compared to FND.

**Table 1:** Characteristics of individuals with factitious disorder or malingering, compared to functional neurological disorder

	FND (N = 143 471)	Factitious disorder (*N* = 5215)			Malingering (*N* = 54 685)		
	*N* (%)	*N* (%)	OR (95% CI)	*P*	*N* (%)	OR (95% CI)	*P*
Age (years): mean (SD)	46.6 (19.3)	52.1 (18.0)	SMD = 0.299	**<0.0001**	48.5 (13.6)	SMD = 0.120	**<0.0001**
Gender							
Female	99 600 (69)	3278 (63)	0.75 (0.70–0.79)	**<0.0001**	21 260 (39)	0.28 (0.27–0.29)	**<0.0001**
Male	40 923 (29)	1765 (34)	1.28 (1.21–1.36)	**<0.0001**	31 947 (58)	3.52 (3.45–3.59)	**<0.0001**
Race							
White	89 623 (62)	3331 (64)	1.06 (1.00–1.13)	0.0394	31 835 (58)	0.84 (0.82–0.85)	**<0.0001**
Black or African American	20 908 (15)	901 (17)	1.22 (1.14–1.32)	**<0.0001**	15 876 (29)	2.40 (2.34–2.46)	**<0.0001**
Asian	2753 (2)	92 (2)	0.92 (0.74–1.13)	0.4231	500 (1)	0.47 (0.43–0.52)	**<0.0001**
American Indian or Alaska Native	877 (0.6)	40 (0.8)	1.26 (0.91–1.73)	0.1591	392 (0.7)	1.17 (1.04–1.32)	0.0085
Native Hawaiian or other Pacific Islander	1267 (0.9)	61 (1.2)	1.33 (1.03–1.72)	0.0313	444 (0.8)	0.92 (0.82–1.02)	0.1258
Other race	5542 (4)	166 (3)	0.82 (0.70–0.96)	0.0122	1004 (2)	0.47 (0.44–0.50)	**<0.0001**
Unknown race	22 501 (16)	624 (12)	0.73 (0.67–0.80)	**<0.0001**	4634 (8)	0.50 (0.48–0.52)	**<0.0001**

Case numbers are provided with percentages in parentheses unless specified otherwise. *P* values (from *t*-tests for comparison of age and *Z*-tests for proportions with a record of categorical variables; highlighted in bold if significant after Bonferroni correction for 10 variables of interest, *P* < 0.005). OR with 95% CI or standardized mean differences (SMD) for the group comparisons are provided.

### Malingering and factitious disorder in FND compared to MS and depression

A total of 145 549 individuals with FND, 237 141 with MS and 2 177 982 with depression were included in the analysis. A total of 1982 FND patients received a malingering record (1.362%) compared to 410 with MS (0.173%) (OR 7.971, 95% CI 7.166–8.867, *P* < 0.0001) and 9246 with depression (0.424%) (OR 3.229, 95% CI 3.077–3.389, *P* < 0.0001).

Nine hundred and twenty-four individuals with FND received a diagnosis of factitious disorder (0.624%) versus 74 with MS (0.031%) (OR 20.468, 95% CI 16.151–25.939, *P* < 0.0001) and 1143 with depression (0.052%) (OR 12.174, 95% CI 11.180–13.307, *P* < 0.0001).

The odds of malingering and factitious disorder diagnoses were still increased after matching the investigated cohorts for age, gender, race, ethnicity and socio-economic status. The associations were even stronger when cases with comorbid FND and MS were excluded from the analysis, whereas excluding cases with comorbid FND and depression had a smaller effect on the odds of recorded feigning (Supplementary Results, Supplementary Tables 1–6).

### Demographic and clinical features of FND with and without a diagnosis of malingering or factitious disorder

**Figure 2:**
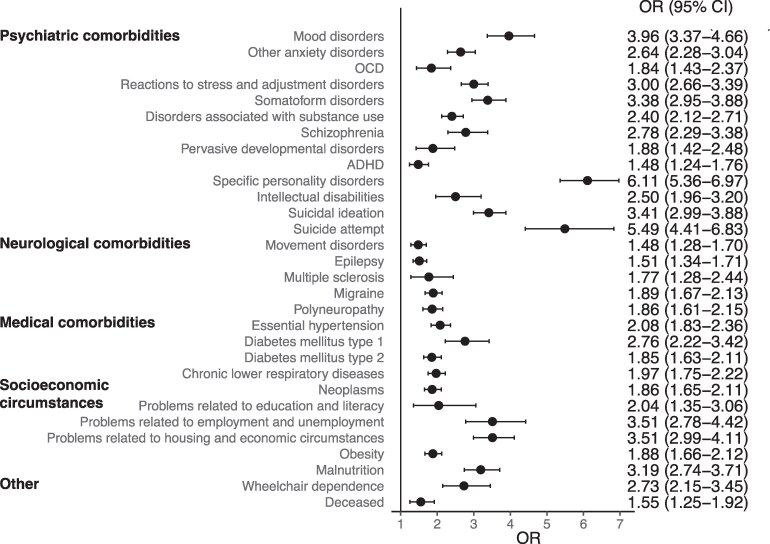
**Odds ratios (OR) with 95% confidence intervals (CI) for comorbidities and socio-economic difficulties in individuals with FND with (*N* = 1061) versus without records of factitious disorder (*N* = 142 773).** Additional data are provided in Table 3. OCD, obsessive-compulsive disorder; ADHD, attention deficit hyperactivity disorder.

**Figure 3:**
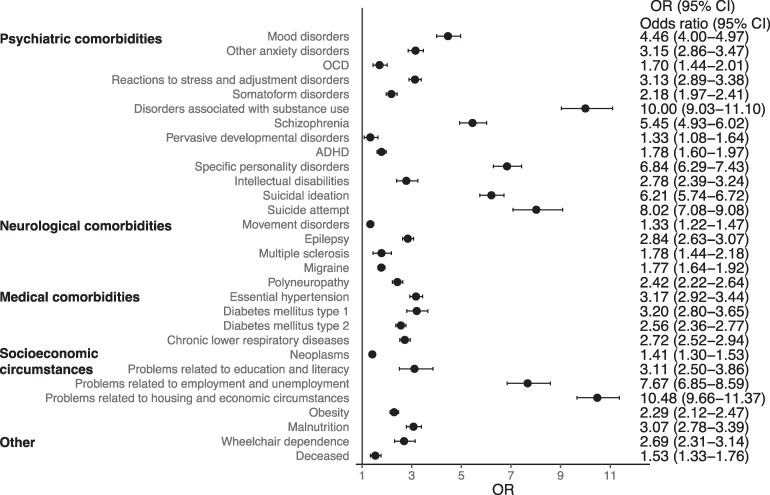
**Odds ratios (OR) with 95% confidence intervals (CI) for comorbidities and socio-economic difficulties in individuals with FND with (*N* = 2607) versus without records of malingering (*N* = 142 773).** Additional data are provided in Table 3. OCD, obsessive-compulsive disorder; ADHD, attention deficit hyperactivity disorder.

In all three groups, females comprised the majority of cases (Table 2). Compared to FND without records of malingering or factitious disorder, individuals with FND and a record of malingering were more frequently male. There were no differences in gender distribution between the group with FND and factitious disorder compared to FND without malingering or factitious disorder. Black or African American race was more commonly associated with malingering, while individuals with factitious disorder were more often White. Higher rates of psychiatric comorbidities, as well as suicidal ideation and suicide attempts, were observed in groups with a record of malingering or factitious disorder compared to those with only FND (Table 3). Similarly, several neurological and medical conditions were more common in these two groups, as well as higher rates of obesity, malnutrition and records of psychosocial and socio-economic difficulties. A greater proportion of individuals with a diagnosis of malingering or factitious disorder required wheelchair use. Mortality was increased in FND with malingering as well as in FND with factitious disorder compared to FND without those records (Figs. 2 and 3).

**Table 2:** Demographic characteristics and clinical presentations of individuals with FND and a record of factitious disorder or malingering, compared to those without these records (only FND)

	Only FND (*N* = 142 773)	FND with factitious disorder (*N* = 1061)			FND with malingering (*N* = 2607)		
	*N* (%)	*N* (%)	OR (95% CI)	*P*	*N* (%)	OR (95% CI)	*P*
Age (years): mean (SD)	46.3 (19.4)	48.9 (18.6)	SMD = 0.137	**<0.0001**	46.5 (13.6)	SMD = 0.013	0.5818
Gender							
Female	99 534 (70)	758 (71)	1.09 (0.95–1.24)	0.2225	1465 (56)	0.56 (0.52–0.60)	**<0.0001**
Male	40 402 (28)	277 (26)	0.90 (0.78–1.03)	0.1144	1050 (40)	1.71 (1.58–1.85)	**<0.0001**
Race							
White	88 996 (62)	714 (67)	1.24 (1.09–1.41)	**0.0009**	1648 (63)	1.04 (0.96–1.13)	0.3578
Black or African American	20 991 (15)	143 (13)	0.90 (0.76–1.08)	0.2617	652 (25)	1.93 (1.77–2.12)	**<0.0001**
Asian	2799 (2)	28 (3)	1.36 (0.93–1.98)	0.1126	27 (1)	0.52 (0.36–0.76)	**0.0007**
American Indian or Alaska Native	858 (0.6)	10 (1)	1.57 (0.84–2.94)	0.1524	32 (1.0)	2.06 (1.44–2.93)	**<0.0001**
Native Hawaiian or other Pacific Islander	1226 (0.9)	12 (1)	1.32 (0.75–2.34)	0.3387	32 (1.2)	1.43 (1.01–2.04)	0.0439
Other race	5109 (4)	37 (3)	0.97 (0.70–1.35)	0.8735	43 (2)	0.45 (0.33–0.61)	**<0.0001**
Unknown race	22 794 (16)	121 (11)	0.68 (0.56–0.82)	**<0.0001**	173 (7)	0.37 (0.32–0.44)	**<0.0001**
FND subtype							
Conversion disorder with motor symptom or deficit	45 951 (32)	294 (28)	0.81 (0.71–0.92)	0.0019	713 (27)	0.79 (0.73–0.87)	**<0.0001**
Conversion disorder with seizures or convulsions	63 270 (44)	430 (41)	0.86 (0.76–0.97)	0.0133	1457 (56)	1.59 (1.47–1.72)	**<0.0001**
Conversion disorder with sensory symptom or deficit	21 386 (15)	139 (13)	0.86 (0.72–1.02)	0.0875	368 (14)	0.93 (0.83–1.04)	0.2208
Conversion disorder with mixed symptom presentation	10 986 (8)	113 (11)	1.43 (1.18–1.74)	**0.0003**	153 (6)	0.75 (0.63–0.88)	**0.0005**
Other dissociative and conversion disorders	14 013 (10)	304 (29)	3.69 (3.23–4.22)	**<0.0001**	205 (8)	0.78 (0.68–0.91)	**0.0009**
Dissociative and conversion disorder, unspecified	32 178 (23)	334 (31)	1.58 (1.39–1.80)	**<0.0001**	864 (33)	1.70 (1.57–1.85)	**<0.0001**

Case counts are provided with percentages in parentheses unless specified otherwise. *P* values for group comparisons (from *t*-tests for comparison of age and *Z*-tests for proportions of categorical variables) are highlighted in bold if significant after Bonferroni correction for 46 variables of interest (*P* < 0.0011). OR with 95% CI or standardised mean differences (SMD) for the comparisons are provided.

**Table 3:** Comorbidities and socio-economic difficulties of individuals with FND and a record of factitious disorder or malingering, compared to those without these records (only FND)

	Only FND (*N* = 142 773)	FND with factitious disorder (*N* = 1061)			FND with malingering (*N* = 2607)		
	*N* (%)	*N* (%)	OR (95% CI)	*P*	*N* (%)	OR (95% CI)	*P*
Psychiatric comorbidities							
Mood disorders	79 886 (56)	885 (83)	3.96 (3.37–4.66)	**<0.0001**	2216 (85)	4.46 (4.00–4.97)	**<0.0001**
Other anxiety disorders	79 729 (56)	816 (77)	2.64 (2.28–3.04)	**<0.0001**	2084 (80)	3.15 (2.86–3.47)	**<0.0001**
OCD	4812 (3)	64 (6)	1.84 (1.43–2.37)	**<0.0001**	146 (6)	1.70 (1.44–2.01)	**<0.0001**
Reactions to stress and adjustment disorders	40 269 (28)	574 (54)	3.00 (2.66–3.39)	**<0.0001**	1437 (55)	3.13 (2.89–3.38)	**<0.0001**
Somatoform disorders	13 569 (10)	278 (26)	3.38 (2.95–3.88)	**<0.0001**	486 (19)	2.18 (1.97–2.41)	**<0.0001**
Disorders associated with substance use	47 168 (33)	575 (54)	2.40 (2.12–2.71)	**<0.0001**	2168 (83)	10.00 (9.03–11.10)	**<0.0001**
Schizophrenia	6030 (4)	116 (11)	2.78 (2.29–3.38)	**<0.0001**	505 (19)	5.45 (4.93–6.02)	**<0.0001**
Pervasive developmental disorders	3816 (3)	52 (5)	1.88 (1.42–2.48)	**<0.0001**	92 (4)	1.33 (1.08–1.64)	0.0074
ADHD	14 116 (10)	148 (14)	1.48 (1.24–1.76)	**<0.0001**	425 (16)	1.78 (1.60–1.97)	**<0.0001**
Specific personality disorders	10 142 (7)	338 (32)	6.11 (5.36–6.97)	**<0.0001**	895 (34)	6.84 (6.29–7.43)	**<0.0001**
Intellectual disabilities	3858 (3)	69 (7)	2.50 (1.96–3.20)	**<0.0001**	187 (7)	2.78 (2.39–3.24)	**<0.0001**
Suicidal ideation	16 894 (12)	333 (31)	3.41 (2.99–3.88)	**<0.0001**	1185 (45)	6.21 (5.74–6.72)	**<0.0001**
Suicide attempt	2398 (2)	91 (9)	5.49 (4.41–6.83)	**<0.0001**	314 (12)	8.02 (7.08–9.08)	**<0.0001**
Neurological comorbidities							
Movement disorders	24 590 (17)	249 (23)	1.48 (1.28–1.70)	**<0.0001**	567 (23)	1.34 (1.22–1.47)	**<0.0001**
Epilepsy	45 236 (32)	437 (41)	1.51 (1.34–1.71)	**<0.0001**	1482 (57)	2.84 (2.63–3.07)	**<0.0001**
Multiple sclerosis	3010 (2)	39 (4)	1.77 (1.28–2.44)	**0.0004**	96 (4)	1.78 (1.44–2.18)	**<0.0001**
Migraine	39 553 (28)	445 (42)	1.89 (1.67–2.13)	**<0.0001**	1054 (40)	1.77 (1.64–1.92)	**<0.0001**
Polyneuropathy	19 118 (13)	237 (22)	1.86 (1.61–2.15)	**<0.0001**	710 (27)	2.42 (2.22–2.64)	**<0.0001**
Medical comorbidities							
Essential hypertension	54 127 (38)	594 (56)	2.08 (1.83–2.36)	**<0.0001**	1719 (66)	3.17 (2.92–3.44)	**<0.0001**
Diabetes mellitus type 1	4700 (3)	91 (9)	2.76 (2.22–3.42)	**<0.0001**	256 (10)	3.20 (2.80–3.65)	**<0.0001**
Diabetes mellitus type 2	27 954 (20)	330 (31)	1.85 (1.63–2.11)	**<0.0001**	1000 (38)	2.56 (2.36–2.77)	**<0.0001**
Chronic lower respiratory diseases	46 353 (32)	516 (49)	1.97 (1.75–2.22)	**<0.0001**	1478 (57)	2.72 (2.52–2.94)	**<0.0001**
Neoplasms	38 793 (27)	435 (41)	1.86 (1.65–2.11)	**<0.0001**	899 (34)	1.41 (1.30–1.53)	**<0.0001**
Diagnoses related to socio-economic circumstances							
Problems related to education and literacy	1604 (1)	24 (2)	2.04 (1.35–3.06)	**0.0005**	89 (3)	3.11 (2.50–3.86)	**<0.0001**
Problems related to employment and unemployment	3200 (2)	79 (7)	3.51 (2.78–4.42)	**<0.0001**	390 (15)	7.67 (6.85–8.59)	**<0.0001**
Problems related to housing and economic circumstances	8262 (6)	188 (18)	3.51 (2.99–4.11)	**<0.0001**	1021 (39)	10.48 (9.66–11.37)	**<0.0001**
Obesity	42 261 (30)	468 (44)	1.88 (1.66–2.12)	**<0.0001**	1278 (49)	2.29 (2.12–2.47)	**<0.0001**
Malnutrition	10 306 (7)	211 (20)	3.19 (2.74–3.71)	**<0.0001**	502 (19)	3.07 (2.78–3.39)	**<0.0001**
Other							
Wheelchair dependence	3876 (3)	75 (7)	2.73 (2.15–3.45)	**<0.0001**	182 (7)	2.69 (2.31–3.14)	**<0.0001**
Deceased	8240 (6)	92 (9)	1.55 (1.25–1.92)	**<0.0001**	223 (9)	1.53 (1.33–1.76)	**<0.0001**

ADHD, attention deficit hyperactivity disorder; OCD, obsessive-compulsive disorder.

Case counts are provided with percentages in parentheses unless specified otherwise. *P* values for group comparisons (from *t*-tests for comparison of age and *Z*-tests for proportions of categorical variables) are highlighted in bold if significant after Bonferroni correction for 46 variables of interest (*P* < 0.0011). OR with 95% CI are also reported.

### Comorbidities associated with stigma in FND with and without a malingering diagnosis

**Figure 4:**
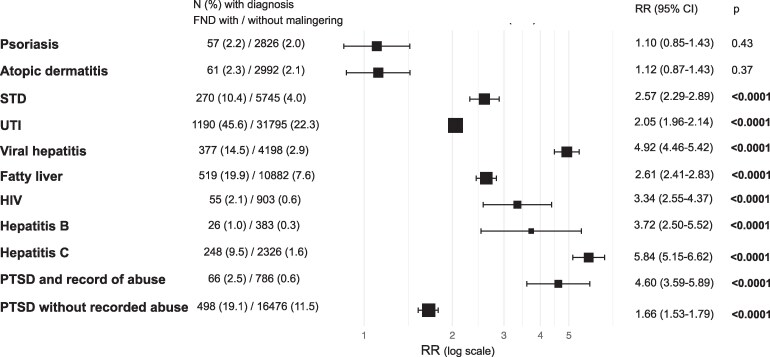
**Relative risk (RR) of a record of malingering in individuals with FND, stratified by comorbid conditions.** Forest plots display RR estimates (with 95% CI) and corresponding *P* values. RRs were calculated from event counts using log-transformed RR formulas and Wald confidence intervals, by comparing the number of individuals with the outcome of interest (a given comorbid condition) and the total number of cases in each cohort: individuals with FND and a record of malingering (*N* = 2607) versus individuals with FND without a record of malingering of factitious disorder (*N* = 142 773). STD, sexually transmitted diseases; UTI, urinary tract infections; PTSD, post-traumatic stress disorder.

The RR of a malingering diagnosis was not increased in individuals with visible skin conditions (Fig. 4). All other examined conditions were associated with an increased RR of a malingering record. Individuals with STD or viral hepatitis carried a higher risk compared to those with UTI or non-alcoholic fatty liver disease, respectively. HIV, hepatitis B and C were associated with an increased risk of a malingering diagnosis. Additionally, the RR of malingering was higher in individuals with PTSD and a history of abuse compared to those without recorded abuse.

### Trends in malingering records in FND over time

**Figure 5:**
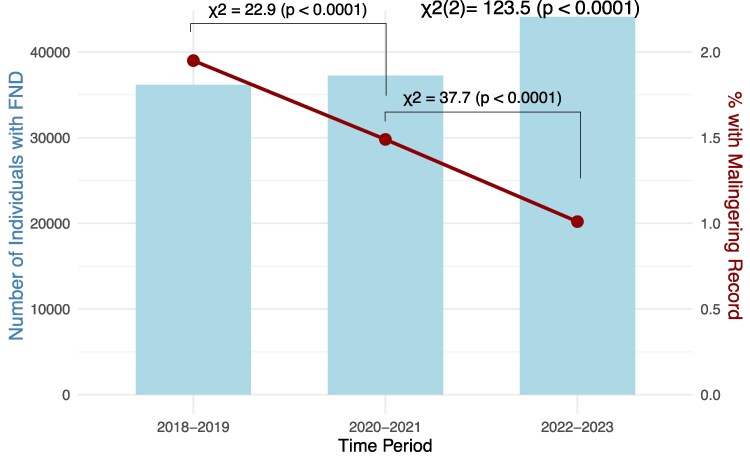
**Malingering records in FND over time.** The number of individuals with a recorded diagnosis of FND is shown for each time period, together with the proportion (%) of those with a record of malingering within one year before or after an FND diagnosis. Chi-squared tests of independence were performed to assess if the proportion of malingering records differed between time periods (the analysis included 36 177 individuals with a recorded diagnosis of FND in 2018–2019, 37 255 in 2020–2021, and 44 112 in 2022–2023).

Among 36 177 patients who had a recorded diagnosis of FND in 2018–2019, 705 (1.95%) also received a diagnosis of malingering within a year before or after. In the 2020–2021 cohort, 554 of 37 255 patients (1.49%) received a malingering record, compared to 445 of 44 112 FND cases (1.01%) in the 2022–2023 cohort. The proportion of cases with a record of malingering differed significantly between time periods (χ^2^(2) = 123.45, *P* < 0.0001) (Fig. 5).

## Discussion

Over a 10-year period within a large international electronic health record database, 2.2% of individuals diagnosed with FND also received a record of malingering or, less commonly, factitious disorder or both. These diagnoses were more frequent in individuals with FND compared to those with MS or depression. Demographic and clinical differences were observed: factitious disorder was more prevalent among White FND patients, whereas male gender, Black or African American race and seizure presentations were more frequent in records of malingering. Individuals with FND and a record of malingering or factitious disorder had higher rates of medical, psychiatric and neurological comorbidities, higher rates of wheelchair use, and a higher rate of recorded socio-economic difficulties. Suicidal ideation and attempts, as well as mortality rates, were also elevated in these subgroups compared to individuals with FND alone.

The prevalence of feigning in clinical practice remains unclear, as it depends on the specific context. In forensic or medicolegal settings, estimates range from 15% to over 50%.^7,31-34^ In clinical practice, rates are likely lower but under-recorded in electronic health records due to under-recognition or deliberate omission.^18^ Many individuals with FND feel that clinicians question the authenticity of their symptoms.^35,36^ The low rates observed in our data likely reflect the rarity of formal diagnoses of feigning rather than the absence of perceived disbelief in clinical encounters.

While symptom exaggeration and feigning can occur in various conditions, the records of malingering and factitious disorder were more common in FND compared to MS or depression, possibly due to several factors. Potential biases may lead clinicians to over-suspect feigning in FND. Further, FND can phenotypically resemble factitious disorder or malingering, and shared risk factors, such as past adversity or socio-economic hardship, can increase their co-occurrence. Alternatively, FND may sometimes be used as a more acceptable label than malingering or factitious disorder, even in cases of misdiagnosis.^37^

Several demographic and clinical variables were associated with a record of malingering or factitious disorder in individuals with FND. While females were more prevalent in all groups, the proportion of males was higher in those diagnosed with malingering. In contrast, gender distribution remained unchanged in FND with a co-diagnosis of factitious disorder, consistent with previous reports.^25,38^ While TriNetX is a global network, most cases originate from the USA, which is an important consideration when interpreting race-related findings. In comparisons of FND cases with versus without records malingering or factitious disorder, Black individuals were more commonly diagnosed with malingering, while factitious disorder was more common among White individuals. Similarly, the over-representation of Black individuals in malingering diagnoses persisted when the analysis was not restricted to those with a co-diagnosis of FND. Gender and racial distributions are comparable to those reported in previous studies of malingering and factitious disorder.^25,39^ The observed demographic differences between groups do not show the kind of monotonic trends that would support a continuum from FND to factitious disorder to malingering,^40^ but suggest that, in electronic health records at least, these diagnoses represent relatively discrete categories.

Our results align with previous studies examining malingering diagnoses in hospital and emergency settings, which have suggested that bias and racism may contribute to the disproportionate application of the malingering label to Black or African American patients and, in turn, overdiagnosis of this condition in this racial group.^17,25^ Similar racial disparities in other mental health diagnoses, such as higher rates of psychotic disorders and conduct disorder versus attention-deficit hyperactivity disorder in certain groups,^41,42^ likely reflect broader structural biases in healthcare. The association between race and deprivation is critical, as individuals facing socio-economic hardship may be perceived as having an external gain for illness. Such diagnostic patterns suggest a concerning shift from comprehensive biopsychosocial frameworks toward reductive views that characterize the experiences of individuals from minority groups as ‘manipulative’, thereby undermining the validity of their reported symptoms and narratives.

Differences in FND phenotype were noted between the groups examined. Functional/dissociative seizures were more common in FND with malingering, whereas motor symptoms were less common. Notably, seizures may pose a challenge for diagnosing malingering, which usually relies on demonstrating marked discrepancies between reported and observed behaviour or video surveillance.^37^ Conversely, frequent emergency presentations for seizures^43,44^ may increase staff exasperation and the possibility of malingering being recorded. Further, seizure semiology may influence clinicians’ interpretation, with atypical presentations prompting suspicion of malingering. Our finding of higher rates of problems related to education and intellectual disability in those with malingering again raises a possible clinician bias, but also suggests a potential for overdiagnosis of feigning in individuals with simpler functional seizure presentations which appear to arise more clearly from the voluntary motor sytem. Functional seizures are more prevalent among those with lower IQ or intellectual disabilities,^45,46^ and may appear more suggestible or more clearly inconsistent with epilepsy in this group, increasing the risk of a malingering label. These hypotheses warrant further investigation.

Neurological and psychiatric comorbidities, including somatic symptoms, suicidal ideation and suicide attempts, were more frequent in FND with malingering or factitious disorder than in FND alone. Effect sizes were largest for substance abuse disorders in cases of malingering and personality disorders in factitious disorder. Given the substantial overlap between psychiatric comorbidities (e.g. personality disorders being more common in FND, malingering and factitious disorder than in the general population),^47^ the diagnosis of any of these disorders at an individual level should not be based on the presence of a psychiatric diagnosis such as a personality disorder. The opposite also applies, namely that cases of malingering may remain unrecognized in individuals without these comorbidities. Nonetheless, the observed pattern of psychiatric comorbidities suggests risk factors that may contribute to the development of factitious disorder and malingering. Previous studies examining psychiatric comorbidity in individuals with factitious disorder and malingering (outside the context of comorbid FND) have likewise reported high rates of psychiatric comorbidity, with mood disorders being the most frequent.^17,48,49^

Neurological comorbidities were common across all three groups examined. Some of these may reflect diagnostic errors. For example, epilepsy was recorded in 31% of individuals with FND without recorded feigning, exceeding the 22% reported in a meta-analysis.^50^ Neurological diagnoses are even more frequent among those with records of factitious disorder and malingering, which may reflect either feigned conditions or genuinely increased rates of neurological comorbidity.

Interestingly, medical comorbidities including hypertension, diabetes and neoplasms, were also more common in FND cases with records of feigning. In some cases, these diagnoses may reflect a history of feigned symptoms or deliberate misreporting of historical medical information. However, in factitious disorder, the experience of an illness that is not feigned, especially in childhood, may provide an initial experience of care and attention within the healthcare system, serving as a conditioning factor that later contributes to factitious behaviour.^51^ Pre-existing health problems may also be aggravated in individuals with factitious disorder.^49^ An alternative explanation is that these individuals have increased rates of genuine medical comorbidities, resulting in greater healthcare utilization and, in turn, a higher likelihood of being labelled as malingering. Repeated medical encounters could therefore be a potential driver of the elevated risk of receiving such a label. A combination of these explanations is possible.

While increased rates of codes indexing socio-economic adversity in malingering may reflect presumed external gain, socio-economic difficulties were also more common in factitious disorder, where such gain is not a defining feature. Factitious behaviour may, however, serve as a maladaptive coping mechanism in response to life stressors and adversity.^49^ Furthermore, opposing diagnoses of obesity and malnutrition were more frequently recorded in individuals with co-diagnoses of malingering or factitious disorder than in those with FND alone, perhaps also reflecting the broader impact of socio-economic deprivation on health. However, potential bias may play a role in attributing feigning-related diagnoses, particularly in the presence of social disadvantage.^21,52^

We explored potential bias in diagnostic patterns further by comparing rates of medical diagnoses associated with significant stigma. Such bias could manifest as overdiagnosis of feigning in individuals with unrelated, stigmatized conditions. While the RR of a malingering record was not elevated in visible skin diseases, it was higher for viral hepatitis compared to non-alcoholic fatty liver disease, and also increased in HIV and STD. It was also greater among PTSD cases with recorded history of abuse compared to those without. This dataset does not allow for an investigation of a possible mediating effect of other covariates, e.g. records of abuse or viral diseases being mediated by mental health diagnoses, socio-economic circumstances or criminal behaviour. Nevertheless, the findings suggest that bias may influence some clinical assessments,^26^ and that accumulating various layers of stigma might contribute to a diagnosis of feigning.^53^

Our unconscious biases may shape our perceptions as clinicians, influence our behaviour and interactions with patients and affect clinical decision-making.^54,55^ Cultivating awareness of our own assumptions, as well as those that may have influenced patients’ previous clinical interactions, can help validate the experience of people with FND and alleviate their stigma. Additionally, stereotypes may inadvertently play a role in medical education.^54,55^ This may be particularly applicable to FND, where a knowledge gap among healthcare professionals and significant regional disparities have been reported.^56^ The observed decline in the malingering diagnoses over recent years could provide a source of optimism. However, it might also be explained by clinicians’ increasing reluctance to ‘diagnose’ malingering. At the same time, underdiagnosis of feigning may also be a concern, as establishing intentional deception or clear discrepancies between reported and observed function requires investigative approaches rarely feasible in clinical encounters.^4,37^

That said, investigating clinician bias using electronic health records has clear limitations, especially the lack of more nuanced insights into individual clinical contexts and the absence of data on clinician characteristics, such as education, speciality and experience. The accuracy of coding is similarly unclear and may be influenced by administrative factors. However, compared to previous studies on the role of bias in malingering records, our large dataset allowed for a more fine-grained analysis of additional risks associated with these diagnoses in the context of FND. A limitation of the study stems from the ICD-10-based definition of malingering, where it is coded as a dichotomous variable. It has been argued that malingering can be viewed as a continuum of behaviours, ranging from symptom exaggeration to invention of symptoms and signs where none exist.^13,57^ While the current study was unable to capture this multifaceted construct, examining whether a diagnostic label is applied to a behaviour provides a suitable means to study the topic of malingering, as well as possible prejudice occurring in clinical settings, as a recorded diagnosis reflects a high level of diagnostic certainty.

The significant proportions of neurological, medical and psychiatric comorbidities in FND with or without feigning point toward a high degree of clinical complexity in these individuals. Many medical settings are ill-equipped to provide the comprehensive level of care required for FND. It is plausible that many individuals with FND undergo multiple clinical examinations because the prevailing models of medical management do not adequately address their needs.^58^ Misattributing this behaviour to ‘doctor shopping’ and feigning, rather than a reasonable response to unaddressed severe symptoms that are not acknowledged or treated, risks causing further harm by reinforcing stigma and further limiting access to treatment.^4,59^ Raising public awareness of FND and improving education across all healthcare disciplines are essential.

In conclusion, FND is rarely accompanied by recorded diagnoses of malingering or factitious disorder, but more commonly than in MS, a comparable medical condition. A co-diagnosis of malingering or factitious disorder is associated with higher rates of psychiatric, medical and neurological comorbidities, as well as increased mortality. The increased risk of a malingering record, linked to race, socio-economic adversity and certain medical conditions, suggests a possible role of clinician bias in some cases. However, we observed more diagnoses as well as declining rates of recorded malingering in FND over recent years, possibly reflecting growing awareness and improved understanding of the disorder.

## Supplementary Material

fcaf490_Supplementary_Data

## Data Availability

TriNetX returned cohort characteristics as csv files, which we archived. They are available to share upon reasonable request. The inclusion criteria used, as specified in Materials and methods and Supplementary Methods, would allow other researchers to identify similar cohorts. However, TriNetX is a dynamic platform with continuously updated data. Therefore, exact case counts may vary over time. The R code used for parts of the statistical analysis in this study is available on GitHub (https://github.com/RokBerlot/Clinical-and-demographic-associations-of-recorded-feigning-in-functional-neurological-disorder).
