# Supplementary material for: Clinical and demographic associations of recorded feigning in functional neurological disorder
Source: Brain Commun. 2025 Dec 12;8(1):fcaf490. doi: 10.1093/braincomms/fcaf490 (PMC12776019; doi:10.1093/braincomms/fcaf490)
Supplement: fcaf490_Supplementary_Data [file fcaf490_supplementary_data.docx]

**SUPPLEMENTARY MATERIAL**

This appendix forms part of the submission of the manuscript:

Berlot R, Pollak TA, Asan L, Stanton B, Nicholson TR, Edwards MJ, Kanaan RA. Clinical and Demographic Associations of Recorded Feigning in Functional Neurological Disorder. Submitted to *Brain Communications* in October 2025.

**Table of Contents**

*Supplementary Methods*.........................................................................................................................................2

The TriNetX network.............................................................................................................................................2

Cohort definition....................................................................................................................................................2

Definition of variables of interest..........................................................................................................................3

Malingering and factitious disorder as outcomes following a diagnosis of FND and MS....................................4

Malingering and factitious disorder as outcomes following a diagnosis of FND and depression.........................4

*Supplementary Results*...........................................................................................................................................5

Malingering and factitious disorder outcomes in matched cohorts of FND and MS.............................................5

Malingering and factitious disorder outcomes in matched cohorts of FND and depression..................................5

Supplementary Table 1..........................................................................................................................................6

Supplementary Table 2..........................................................................................................................................7

Supplementary Table 3..........................................................................................................................................8

Supplementary Table 4..........................................................................................................................................9

Supplementary Table 5.........................................................................................................................................10

Supplementary Table 6.........................................................................................................................................11

**SUPPLEMENTARY METHODS**

**The TriNetX network**

This section provides additional information on the TriNetX network, detailing the data sources, its structure, the quality control measures of the dataset, its advantages and disadvantages.

To comply with legal and ethical standards, the individual contributing healthcare organisations remain anonymous, and their individual contributions to the dataset cannot be assessed. The database encompasses structured clinical data, which represents real-world clinical data. The data are standardised using recognised clinical terminologies, which include demographics, diagnoses, procedures, and measurements. Variables such as education or occupational status are not represented unless proxies are provided using the above codes. Updates from contributing healthcare organisations are continuous; consequently, the number of patients included and available for analysis is updated continuously. To maintain privacy, contributing institutions remain anonymous. The data are stored on a TriNetX appliance, which may be physical servers at the institution's data centre or a virtual hosted system.

In response to centralised queries, results are collected and aggregated. Data are standardised using recognised clinical terminologies, which include demographics (coded according to HL7 standards) and diagnoses (represented by ICD-10-CM, the International Classification of Diseases, Tenth Revision, Clinical Modification), procedures and measurements. The data analysed are secondary data, do not involve intervention or interaction with human subjects, and have been de-identified in line with the de-identification standard outlined in Section §164.514(b)(1) of the HIPAA Privacy Rule (http://trinetx.com).

TriNetX includes data from both insured and uninsured individuals, offering a broader view of clinical diagnoses than insurance claims databases. Unlike survey-based data, EHR data reflect actual diagnostic rates among healthcare users, providing a more accurate account of the burden of specific diagnoses within the system. Patients often receive care across multiple healthcare organisations, and if some are not part of the network, their records may be incomplete. However, this limitation is not unique to TriNetX and similarly affects single-centre retrospective studies. Using a multi-centre network of healthcare organisations may help mitigate this issue.

TriNetX employs a structured quality control protocol to ensure that the data meet predefined standards and are aligned with required formats, completeness, and plausibility. Each assessment domain has specific predefined metrics, and sites must meet the required criteria before their data are included. Records lacking essential fields or containing only demographic information are excluded. Data are monitored for consistency over time, and software is subject to rigorous validation using synthetic test data and cross-verification between independent codebases in different languages (e.g. R and Python). All code is independently reviewed and tested to ensure reliability. Additional information on the strategy used is available on the network website (http://trinetx.com).

**Cohort definition**

The diagnosis of functional neurological disorder (FND) was defined as any of the following ICD-10 categories: Conversion disorder with motor symptom or deficit (F44.4), Conversion disorder with seizures or convulsions (F44.5), Conversion disorder with sensory symptom or deficit (F44.6), Conversion disorder with mixed symptom presentation (F44.7), Other dissociative and conversion disorders (F44.89), Dissociative and conversion disorder, unspecified (F44.9). These codes are most commonly used for the typical phenotypes of FND: functional motor disorder, functional/dissociative seizures, functional sensory disorder, FND with mixed symptoms, and other or unspecified FND. We excluded other F44 diagnostic category codes, such as dissociative fugue, amnesia, stupor, and identity disorder, as these are not typically used for the most common FND phenotypes.

In addition, cohorts with a new record of 'Malingerer (conscious simulation)' (Z76.5) or 'Factitious disorder imposed on self' (F68.1) in the same time period were constructed.

Cases satisfying inclusion criteria, specified above, between 1 January 2015 and 31 December 2024 were included in the analyses. Cases with a record of a diagnosis from the group of conversion and dissociative disorders (F44), other disorders of adult personality and behaviour (F68), and malingerer (conscious simulation) (Z76.5) before 1 January 2015 were excluded.

Individuals with a new diagnosis of multiple sclerosis (MS) (G35) recorded between 1 January 2015 and 31 December 2024 formed the control cohort for the case-control analysis.

**Definition of variables of interest**

We assessed the following demographic variables, psychiatric, neurological, and medical comorbidities, records of socioeconomic adversity, indicators of disease severity, and mortality in the comparison between FND cases with versus without records of malingering and factitious disorder:

1) **Age**

2) **Gender,** encoded as two separate dichotomous variables: Female and Male

3) **Race,** encoded as seven separate dichotomous variables: White, Black or African American, Asian, American Indian or Alaska Native, Native Hawaiian or Other Pacific Islander, Other Race, Unknown Race.

4) **Mood (affective) disorders**, encoded as a dichotomous variable (F30-F39)

5) **Other anxiety disorders**, encoded as a dichotomous variable (F41)

6) **Obsessive-compulsive disorder**, encoded as a dichotomous variable (F)

7) **Reaction to stress and adjustment disorders**, encoded as a dichotomous variable (F43)

8) **Somatoform disorders**, encoded as a dichotomous variable (F45)

9) **Disorders associated with substance use**, encoded as a dichotomous variable (F10-F19)

10) **Schizophrenia**, encoded as a dichotomous variable (F20)

11) **Pervasive developmental disorders**, encoded as a dichotomous variable (F84)

12) **Attention deficit hyperactivity disorder**, encoded as a dichotomous variable (F90)

13) **Specific personality disorders**, encoded as a dichotomous variable (F84)

14) **Intellectual disabilities**, encoded as a dichotomous variable (F70-F79)

15) **Suicidal ideation**, encoded as a dichotomous variable (R45.851)

16) **Suicide attempt**, encoded as a dichotomous variable (T14.91)

17) **Movement disorders**, encoded as a dichotomous variable: Extrapyramidal and movement disorders (G20-G26)

18) **Epilepsy**, encoded as a dichotomous variable: Epilepsy and recurrent seizures (G40)

19) **Multiple sclerosis,** encoded as a dichotomous variable (G35)

20) **Migraine**, encoded as a dichotomous variable (G43)

21) **Polyneuropathy**, encoded as a dichotomous variable: Polyneuropathies and other disorders of the peripheral nervous system (G60-G65)

22) **Essential hypertension,** encoded as a dichotomous variable (I10)

23) **Diabetes mellitus,** encoded as two dichotomous variables: Type 1 (E10) and Type 2 (E11) diabetes mellitus

24) **Chronic lower respiratory diseases,** encoded as a dichotomous variable (J40-J47)

25) **Neoplasms,** encoded as a dichotomous variable (C00-D49)

26) **Problems related to education and literacy,** encoded as a dichotomous variable (Z55)

27) **Problems related to employment and unemployment,** encoded as a dichotomous variable (Z56)

28) **Problems related to housing and economic circumstances,** encoded as a dichotomous variable (Z59)

29) **Obesity,** encoded as a dichotomous variable: Overweight and obesity (E66)

30) **Malnutrition,** encoded as a dichotomous variable (E40-E46)

31) **Dependence on wheelchair,** encoded as a dichotomous variable (Z99.3)

32) **Deceased**

The following variables were identified to examine the role of comorbidities associated with stigma in FND cases with versus without records of malingering:

1) **Psoriasis**, encoded as a dichotomous variable (L40)

2) **Atopic dermatitis**, encoded as a dichotomous variable (L20)

3) **Sexually transmitted diseases**, encoded as a dichotomous variable (A50-A64)

4) **Urinary tract infections**, encoded as a dichotomous variable (N39.0)

5) **Viral hepatitis**, encoded as a dichotomous variable (B15-B19), and additionally as sub-categories with two dichotomous variables: hepatitis B (B18.1) and hepatitis C (B18.2)

6) **Non-alcoholic fatty liver,** encoded as a dichotomous variable (K76.0)

7) **HIV disease,** encoded as a dichotomous variable (B20)

8) **Post-traumatic stress disorder,** encoded as a dichotomous variable (F43.1)

9) **Adult and child abuse, neglect, and other maltreatment, confirmed,** encoded as a dichotomous variable (T74)

**Malingering and factitious disorder as outcomes following a diagnosis of FND versus MS**

The cohort of patients with FND was compared to a contemporaneous cohort with newly diagnosed MS (G35). The diagnoses of the respective conditions were selected as index events. Two outcomes were assessed after this index event: a new diagnosis of malingering and that of factitious disorder. We compared these outcomes in cohorts with FND versus MS, and calculated the odds ratios (OR).

In addition to comparing outcomes in the two unmatched groups, sensitivity analyses were performed.

(i) To ensure group differences were not due to differences in demographic variables, the cohorts of patients with FND and MS were matched before comparison for age at diagnosis, gender (male, female, unknown), race (White, Black or African American, Asian, Native Hawaiian or other Pacific Islander, American Indian or Alaska Native, Other Race, Unknown Race), and ethnicity (Hispanic or Latino, Not Hispanic or Latino, Unknown Ethnicity). A propensity score-matching algorithm integrated into the TriNetX platform was used. The algorithm employs logistic regression to calculate propensity scores based on user-specified covariates. It then applies a greedy nearest-neighbour matching algorithm with a 1:1 ratio to pair cases and controls.

(ii) To account for differences in socioeconomic circumstances between groups with FND and MS, the record of a diagnosis from the Z55-Z65 diagnostic category (Persons with potential health hazards related to socioeconomic and psychosocial circumstances), encoded as a dichotomous variable, was used in the propensity matching algorithm in addition to the demographic variables specified above.

(iii) To eliminate the influence of comorbidity between FND and MS on the results, an additional sensitivity analysis was performed between groups matched for demographic variables in which FND cases were excluded from the MS cohort, and MS cases were excluded from the FND cohort.

Baseline characteristics were adequately balanced, with standardised mean differences below 0.1 for all variables of interest (Supplementary Tables 1-3).

**Malingering and factitious disorder as outcomes following a diagnosis of FND versus depression**

The cohort of patients with FND was compared to a contemporaneous cohort with newly diagnosed recurrent major depressive disorder (F33).

Outcomes were compared in the two unmatched groups, and additional sensitivity analyses were performed, as above, with (i) cohorts matched for age, gender, race, and ethnicity; (ii) additionally matched for records of diagnoses from the Z55-Z65 diagnostic category; and (iii) matched for demographic variables with FND cases excluded from the depression cohort, and depression cases excluded from the FND cohort.

As the sensitivity analyses were performed at a later date (October 2025), and TriNetX is a dynamic network, the number of cases included in the FND cohort differed from the comparison above. Baseline characteristics were well-balanced (Supplementary Tables 4-6).

**SUPPLEMENTARY RESULTS**

**Malingering and factitious disorder outcomes in matched cohorts of FND and MS**

When comparing groups matched for differences in demographic variables (122,049 individuals in each group) (Supplementary Table 1), 1,046 patients with FND received a record of malingering (1.444%), compared to 231 with MS (0.189%) (OR 7.725, 95% CI 6.733-8.862, *p*<.0001). 809 individuals with FND received a diagnosis of factitious disorder (0.663%) compared to 38 with MS (0.031%) (OR 21.425, 95% CI 15.473-29.665, *p*<.0001).

When the two cohorts were additionally matched for differences in socioeconomic and psychosocial circumstances (116,328 individuals in each group) (Supplementary Table 2), 1,762 patients with FND received a record of malingering (0.905%), compared to 186 with MS (0.160%) (OR 5.695, 95% CI 4.872-6.658, *p*<.0001). 672 patients with FND were diagnosed with factitious disorder (0.579%) compared to 37 with MS (0.032%) (OR 18.291, 95% CI 13.136-25.470, *p*<.0001).

When cases with comorbid FND and MS were excluded and groups were matched for demographic variables (age, gender, race, ethnicity) (Supplementary Table 3), 118,928 individuals remained in each group. The odds of malingering were greater in FND (1,711 patients, 1.439%) compared to MS (186 patients, 0.156%) (OR 9.319, 95% CI 8.008-10.843, *p*<.0001). Similarly, the odds of a factitious disorder diagnosis were greater in FND (799 patients, 0.672%) compared to MS (22 patients, 0.018%) (OR 36.557, 95% CI 23.932-55.843, *p*<.0001).

**Malingering and factitious disorder outcomes in matched cohorts of FND and depression**

In the comparison of groups matched for demographic variables (139,476 individuals in each group) (Supplementary Table 4), 1,709 patients with FND received a record of malingering (1.225%), compared to 611 with depression (0.438%) (OR 2.819, 95% CI 2.570-3.093, *p*<.0001). 903 patients with FND received a diagnosis of factitious disorder (0.647%) compared to 83 with depression (0.060%) (OR 10.994, 95% CI 8.739-13.704, *p*<.0001).

After additionally matching for differences in socioeconomic and psychosocial circumstances (139,476 individuals in each group) (Supplementary Table 5), the number of individuals with outcomes of interest in FND group remained unchanged. 614 individuals with depression received a record of malingering (0.440%), (OR 2.806, 95% CI 2.558-3.077, *p*<.0001), and 82 of factitious disorder (0.059%) (OR 11.077, 95% CI 8.835-13.889, *p*<.0001).

After excluding cases with comorbid FND and depression and matching cohorts for demographic variables (age, gender, race, ethnicity) (Supplementary Table 6), 108,753 individuals remained in each group. The odds of malingering were greater in FND (997 patients, 0.481%) compared to depression (367 patients, 0.337%) (OR 2.733, 95% CI 2.424-3.081, *p*<.0001). In addition, the odds of factitious disorder were greater in FND (523 patients, 0.481%) than in depression (48 patients, 0.044%) (OR 10.944, 95% CI 8.142-14.709, *p*<.0001).

**Supplementary Table 1.** Demographic characteristics of patients with functional neurological disorder (Cohort 1) and multiple sclerosis (Cohort 2) before and after matching for demographic variables. SMD: standardised mean difference.

| **Cohort characteristics** | | | | | | | | | **Cohort 1 (N = 142,406) and Cohort 2 (N = 233,521) before propensity score matching** | | | | | **Cohort 1 (N = 122,049) and Cohort 2 (N = 122,049) after propensity score matching** | | | | | |
| --- | --- | --- | --- | --- | --- | --- | --- | --- | --- | --- | --- | --- | --- | --- | --- | --- | --- | --- | --- |
|  | **Demographics** | | | |  |  |  | | |  | | | | | | | | | |
|  |  | Cohort | |  | | | | Mean ± SD | | | Patients | % of Cohort | SMD | | Mean ± SD | Patients | % of Cohort | SMD |  |
|  |  | 1 2 |  | Age at Diagnosis | | | | 41.2 +/- 19.0 48.4 +/- 14.8 | | | 142,406 233,521 | 100% 100% | 0.422 | | 45.0 +/- 17.8 45.7 +/- 16.6 | 122,049 122,049 | 100% 100% | 0.041 |  |
|  |  | 1 2 |  | Female | | | |  | | | 99,375 164,495 | 69.8% 70.4% | 0.014 | |  | 85,758 81,767 | 70.3% 67.0% | 0.071 |  |
|  |  | 1 2 |  | Black or African American | | | |  | | | 21,819 29,014 | 15.3% 12.4% | 0.084 | |  | 17,245 19,126 | 14.1% 15.7% | 0.043 |  |
|  |  | 1 2 |  | Male | | | |  | | | 40,082 59,678 | 28.1% 25.6% | 0.058 | |  | 33,357 37,380 | 27.3% 30.6% | 0.073 |  |
|  |  | 1 2 |  | White | | | |  | | | 91,332 145,944 | 64.1% 62.5% | 0.034 | |  | 77,891 76,666 | 63.8% 62.8% | 0.021 |  |
|  |  | 1 2 |  | American Indian or Alaska Native | | | |  | | | 898 913 | 0.6% 0.4% | 0.034 | |  | 632 723 | 0.5% 0.6% | 0.010 |  |
|  |  | 1 2 |  | Unknown Race | | | |  | | | 19,080 45,491 | 13.4% 19.5% | 0.165 | |  | 18,945 17,656 | 15.5% 14.5% | 0.030 |  |
|  |  | 1 2 |  | Native Hawaiian or Other Pacific Islander | | | |  | | | 1,271 1,579 | 0.9% 0.7% | 0.025 | |  | 982 982 | 0.8% 0.8% | <0.001 |  |
|  |  | 1 2 |  | Other Race | | | |  | | | 5,151 7,762 | 3.6% 3.3% | 0.016 | |  | 4,474 4,369 | 3.7% 3.6% | 0.005 |  |
|  |  | 1 2 |  | Asian | | | |  | | | 2,855 2,818 | 2.0% 1.2% | 0.064 | |  | 1,880 2,527 | 1.5% 2.1% | 0.040 |  |

**Supplementary Table 2.** Demographic characteristics of patients with functional neurological disorder (Cohort 1) and multiple sclerosis (Cohort 2), before and after matching for demographic variables and records of socioeconomic difficulties. SMD: standardised mean difference.

| **Cohort characteristics** | | | | | | | | | **Cohort 1 (N = 142,800) and Cohort 2 (N = 237,859) before propensity score matching** | | | | | **Cohort 1 (N = 116,328) and Cohort 2 (N = 116,328) after propensity score matching** | | | | | |
| --- | --- | --- | --- | --- | --- | --- | --- | --- | --- | --- | --- | --- | --- | --- | --- | --- | --- | --- | --- |
|  | **Demographics** | | | |  |  |  | | |  | | | | | | | | | |
|  |  | Cohort | |  | | | | Mean ± SD | | | Patients | % of Cohort | SMD | | Mean ± SD | Patients | % of Cohort | SMD |  |
|  |  | 1 2 |  | Age at Diagnosis | | | | 41.3 +/- 19.0 48.5 +/- 14.8 | | | 142,800 237,859 | 100% 100% | 0.421 | | 44.9 +/- 18.0 45.7 +/- 16.8 | 116,328 116,328 | 100% 100% | 0.045 |  |
|  |  | 1 2 |  | Female | | | |  | | | 99,364 167,561 | 69.6% 70.4% | 0.019 | |  | 81,494 78,403 | 70.1% 67.4% | 0.057 |  |
|  |  | 1 2 |  | Black or African American | | | |  | | | 21,419 29,045 | 15.0% 12.2% | 0.081 | |  | 16,065 17,238 | 13.8% 14.8% | 0.029 |  |
|  |  | 1 2 |  | Male | | | |  | | | 40,483 60,949 | 28.3% 25.6% | 0.061 | |  | 32,077 35,269 | 27.6% 30.3% | 0.061 |  |
|  |  | 1 2 |  | White | | | |  | | | 90,231 148,961 | 63.2% 62.6% | 0.012 | |  | 73,412 71,638 | 63.1% 61.6% | 0.031 |  |
|  |  | 1 2 |  | American Indian or Alaska Native | | | |  | | | 897 890 | 0.6% 0.4% | 0.036 | |  | 568 738 | 0.5% 0.6% | 0.020 |  |
|  |  | 1 2 |  | Unknown Race | | | |  | | | 20,576 46,877 | 14.4% 19.7% | 0.141 | |  | 19,325 18,443 | 16.6% 15.9% | 0.021 |  |
|  |  | 1 2 |  | Native Hawaiian or Other Pacific Islander | | | |  | | | 1,262 1,555 | 0.9% 0.7% | 0.026 | |  | 927 955 | 0.8% 0.8% | 0.003 |  |
|  |  | 1 2 |  | Other Race | | | |  | | | 5,546 7,652 | 3.9% 3.2% | 0.036 | |  | 4,216 4,728 | 3.6% 4.1% | 0.023 |  |
|  |  | 1 2 |  | Asian | | | |  | | | 2,869 2,879 | 2.0% 1.2% | 0.063 | |  | 1,815 2,588 | 1.6% 2.2% | 0.049 |  |
|  |  | 1 2 |  | Persons with potential health hazards related to socioeconomic and psychosocial circumstances | | | |  | | | 14,193 3,914 | 9.9% 1.6% | 0.361 | |  | 3,760 3,914 | 3.2% 3.4% | 0.007 |  |

**Supplementary Table 3.** Demographic characteristics of patients with functional neurological disorder (Cohort 1) and multiple sclerosis (Cohort 2), after excluding cases with comorbid functional neurological disorder and multiple sclerosis, before and after matching. SMD: standardised mean difference.

| **Cohort characteristics** | | | | | | | | | **Cohort 1 (N = 139,279) and Cohort 2 (N = 230,803) before propensity score matching** | | | | | **Cohort 1 (N = 118,928) and Cohort 2 (N = 118,928) after propensity score matching** | | | | | |
| --- | --- | --- | --- | --- | --- | --- | --- | --- | --- | --- | --- | --- | --- | --- | --- | --- | --- | --- | --- |
|  | **Demographics** | | | |  |  |  | | |  | | | | | | | | | |
|  |  | Cohort | |  | | | | Mean ± SD | | | Patients | % of Cohort | SMD | | Mean ± SD | Patients | % of Cohort | SMD |  |
|  |  | 1 2 |  | Age at Diagnosis | | | | 41.1 +/- 19.0 48.5 +/- 14.8 | | | 139,279 230,803 | 100% 100% | 0.431 | | 45.0 +/- 17.8 45.7 +/- 16.6 | 118,928 118,928 | 100% 100% | 0.042 |  |
|  |  | 1 2 |  | Female | | | |  | | | 96,838 162,295 | 69.5% 70.3% | 0.017 | |  | 83,196 79,426 | 70.0% 66.8% | 0.068 |  |
|  |  | 1 2 |  | Black or African American | | | |  | | | 21,286 28,568 | 15.3% 12.4% | 0.084 | |  | 16,802 18,824 | 14.1% 15.8% | 0.048 |  |
|  |  | 1 2 |  | Male | | | |  | | | 39,569 59,241 | 28.4% 25.7% | 0.062 | |  | 32,881 36,683 | 27.6% 30.8% | 0.070 |  |
|  |  | 1 2 |  | White | | | |  | | | 89,227 144,142 | 64.1% 62.5% | 0.033 | |  | 75,805 74,162 | 63.7% 62.4% | 0.029 |  |
|  |  | 1 2 |  | American Indian or Alaska Native | | | |  | | | 868 886 | 0.6% 0.4% | 0.034 | |  | 569 681 | 0.5% 0.6% | 0.013 |  |
|  |  | 1 2 |  | Unknown Race | | | |  | | | 18,784 45,209 | 13.5% 19.6% | 0.165 | |  | 18,628 17,066 | 15.7% 14.3% | 0.037 |  |
|  |  | 1 2 |  | Native Hawaiian or Other Pacific Islander | | | |  | | | 1,229 1,538 | 0.9% 0.7% | 0.025 | |  | 937 994 | 0.8% 0.8% | 0.005 |  |
|  |  | 1 2 |  | Other Race | | | |  | | | 5,062 7,675 | 3.6% 3.3% | 0.017 | |  | 4,383 4,621 | 3.7% 3.9% | 0.010 |  |
|  |  | 1 2 |  | Asian | | | |  | | | 2,823 2,785 | 2.0% 1.2% | 0.065 | |  | 1,804 2,580 | 1.5% 2.2% | 0.049 |  |

**Supplementary Table 4.** Demographic characteristics of patients with functional neurological disorder (Cohort 1) and recurrent major depressive disorder (Cohort 2) before and after matching for demographic variables. SMD: standardised mean difference.

| **Cohort characteristics** | | | | | | | | | **Cohort 1 (N = 139,476) and Cohort 2 (N = 2,102,926) before propensity score matching** | | | | | **Cohort 1 (N = 139,476) and Cohort 2 (N = 139,476) after propensity score matching** | | | | | |
| --- | --- | --- | --- | --- | --- | --- | --- | --- | --- | --- | --- | --- | --- | --- | --- | --- | --- | --- | --- |
|  | **Demographics** | | | |  |  |  | | |  | | | | | | | | | |
|  |  | Cohort | |  | | | | Mean ± SD | | | Patients | % of Cohort | SMD | | Mean ± SD | Patients | % of Cohort | SMD |  |
|  |  | 1 2 |  | Age at Diagnosis | | | | 41.0 +/- 18.9 43.2 +/- 18.7 | | | 139,476 2,102,926 | 100% 100% | 0.121 | | 41.0 +/- 18.9 41.0 +/- 18.9 | 139,476 139,476 | 100% 100% | <0.001 |  |
|  |  | 1 2 |  | Female | | | |  | | | 99,332 1,424,551 | 71.2% 67.7% | 0.076 | |  | 99,332 99,336 | 71.2% 71.2% | <0.001 |  |
|  |  | 1 2 |  | Black or African American | | | |  | | | 20,382 244,384 | 14.6% 11.6% | 0.089 | |  | 20,382 20,381 | 14.6% 14.6% | <0.001 |  |
|  |  | 1 2 |  | Male | | | |  | | | 40,005 677,154 | 28.7% 32.2% | 0.077 | |  | 40,005 40,007 | 28.7% 28.7% | <0.001 |  |
|  |  | 1 2 |  | White | | | |  | | | 91,145 1,533,939 | 65.3% 72.9% | 0.165 | |  | 91,145 91,148 | 65.3% 65.4% | <0.001 |  |
|  |  | 1 2 |  | American Indian or Alaska Native | | | |  | | | 915 11,440 | 0.7% 0.5% | 0.015 | |  | 915 914 | 0.7% 0.7% | <0.001 |  |
|  |  | 1 2 |  | Unknown Race | | | |  | | | 17,010 166,748 | 12.2% 7.9% | 0.142 | |  | 17,010 17,016 | 12.2% 12.2% | <0.001 |  |
|  |  | 1 2 |  | Native Hawaiian or Other Pacific Islander | | | |  | | | 649 7,148 | 0.5% 0.3% | 0.020 | |  | 649 648 | 0.5% 0.5% | <0.001 |  |
|  |  | 1 2 |  | Other Race | | | |  | | | 6,582 96,416 | 4.7% 4.6% | 0.006 | |  | 6,582 6,579 | 4.7% 4.7% | <0.001 |  |
|  |  | 1 2 |  | Asian | | | |  | | | 2,793 42,851 | 2.0% 2.0% | 0.003 | |  | 2,793 2,790 | 2.0% 2.0% | <0.001 |  |

**Supplementary Table 5.** Demographic characteristics of patients with functional neurological disorder (Cohort 1) and recurrent major depressive disorder (Cohort 2), before and after matching for demographic variables and records of socioeconomic difficulties. SMD: standardised mean difference.

| **Cohort characteristics** | | | | | | | | | **Cohort 1 (N = 139,476) and Cohort 2 (N = 2,102,926) before propensity score matching** | | | | | **Cohort 1 (N = 139,476) and Cohort 2 (N = 139,476) after propensity score matching** | | | | | |
| --- | --- | --- | --- | --- | --- | --- | --- | --- | --- | --- | --- | --- | --- | --- | --- | --- | --- | --- | --- |
|  | **Demographics** | | | |  |  |  | | |  | | | | | | | | | |
|  |  | Cohort | |  | | | | Mean ± SD | | | Patients | % of Cohort | SMD | | Mean ± SD | Patients | % of Cohort | SMD |  |
|  |  | 1 2 |  | Age at Diagnosis | | | | 41.0 +/- 18.9 43.2 +/- 18.7 | | | 139,476 2,102,926 | 100% 100% | 0.121 | | 41.0 +/- 18.9 41.0 +/- 18.9 | 139,476 139,476 | 100% 100% | <0.001 |  |
|  |  | 1 2 |  | Female | | | |  | | | 99,332 1,424,551 | 71.2% 67.7% | 0.076 | |  | 99,332 99,343 | 71.2% 71.2% | <0.001 |  |
|  |  | 1 2 |  | Black or African American | | | |  | | | 20,382 244,384 | 14.6% 11.6% | 0.089 | |  | 20,382 20,378 | 14.6% 14.6% | <0.001 |  |
|  |  | 1 2 |  | Male | | | |  | | | 40,005 677,154 | 28.7% 32.2% | 0.077 | |  | 40,005 40,002 | 28.7% 28.7% | <0.001 |  |
|  |  | 1 2 |  | White | | | |  | | | 91,145 1,533,939 | 65.3% 72.9% | 0.165 | |  | 91,145 91,146 | 65.3% 65.3% | <0.001 |  |
|  |  | 1 2 |  | American Indian or Alaska Native | | | |  | | | 915 11,440 | 0.7% 0.5% | 0.015 | |  | 915 911 | 0.7% 0.7% | <0.001 |  |
|  |  | 1 2 |  | Unknown Race | | | |  | | | 17,010 166,748 | 12.2% 7.9% | 0.142 | |  | 17,010 17,027 | 12.2% 12.2% | <0.001 |  |
|  |  | 1 2 |  | Native Hawaiian or Other Pacific Islander | | | |  | | | 649 7,148 | 0.5% 0.3% | 0.020 | |  | 649 643 | 0.5% 0.5% | 0.001 |  |
|  |  | 1 2 |  | Other Race | | | |  | | | 6,582 96,416 | 4.7% 4.6% | 0.006 | |  | 6,582 6,584 | 4.7% 4.7% | <0.001 |  |
|  |  | 1 2 |  | Asian | | | |  | | | 2,793 42,851 | 2.0% 2.0% | 0.003 | |  | 2,793 2,787 | 2.0% 2.0% | <0.001 |  |

**Supplementary Table 6.** Demographic characteristics of patients with functional neurological disorder (Cohort 1) and recurrent major depressive disorder (Cohort 2), after excluding cases with comorbid functional neurological disorder and depression, before and after matching. SMD: standardised mean difference.

| **Cohort characteristics** | | | | | | | | | **Cohort 1 (N = 108,753) and Cohort 2 (N = 2,068,505) before propensity score matching** | | | | | **Cohort 1 (N = 108,753) and Cohort 2 (N = 108,753) after propensity score matching** | | | | | |
| --- | --- | --- | --- | --- | --- | --- | --- | --- | --- | --- | --- | --- | --- | --- | --- | --- | --- | --- | --- |
|  | **Demographics** | | | |  |  |  | | |  | | | | | | | | | |
|  |  | Cohort | |  | | | | Mean ± SD | | | Patients | % of Cohort | SMD | | Mean ± SD | Patients | % of Cohort | SMD |  |
|  |  | 1 2 |  | Age at Diagnosis | | | | 41.1 +/- 19.2 43.3 +/- 18.7 | | | 108,753 2,068,505 | 100% 100% | 0.116 | | 41.1 +/- 19.2 41.1 +/- 19.2 | 108,753 108,753 | 100% 100% | <0.001 |  |
|  |  | 1 2 |  | Female | | | |  | | | 75,441 1,397,857 | 69.4% 67.6% | 0.039 | |  | 75,441 75,445 | 69.4% 69.4% | <0.001 |  |
|  |  | 1 2 |  | Black or African American | | | |  | | | 15,999 239,535 | 14.7% 11.6% | 0.093 | |  | 15,999 16,000 | 14.7% 14.7% | <0.001 |  |
|  |  | 1 2 |  | Male | | | |  | | | 33,209 669,472 | 30.5% 32.4% | 0.039 | |  | 33,209 33,211 | 30.5% 30.5% | <0.001 |  |
|  |  | 1 2 |  | White | | | |  | | | 68,745 1,508,860 | 63.2% 72.9% | 0.210 | |  | 68,745 68,744 | 63.2% 63.2% | <0.001 |  |
|  |  | 1 2 |  | American Indian or Alaska Native | | | |  | | | 661 11,136 | 0.6% 0.5% | 0.009 | |  | 661 661 | 0.6% 0.6% | <0.001 |  |
|  |  | 1 2 |  | Unknown Race | | | |  | | | 15,311 164,798 | 14.1% 8.0% | 0.196 | |  | 15,311 15,318 | 14.1% 14.1% | <0.001 |  |
|  |  | 1 2 |  | Native Hawaiian or Other Pacific Islander | | | |  | | | 537 7,025 | 0.5% 0.3% | 0.024 | |  | 537 533 | 0.5% 0.5% | 0.001 |  |
|  |  | 1 2 |  | Other Race | | | |  | | | 5,155 94,815 | 4.7% 4.6% | 0.007 | |  | 5,155 5,155 | 4.7% 4.7% | <0.001 |  |
|  |  | 1 2 |  | Asian | | | |  | | | 2,345 42,336 | 2.2% 2.0% | 0.008 | |  | 2,345 2,342 | 2.2% 2.2% | <0.001 |  |
